# Changes in the quality of life of early breast cancer patients and comparison with the normative Slovenian population

**DOI:** 10.2478/raon-2023-0019

**Published:** 2023-06-21

**Authors:** Cvetka Grasic Kuhar, Tjasa Gortnar Cepeda, Christian Kurzeder, Marcus Vetter

**Affiliations:** Institute of Oncology Ljubljana, Department Medical Oncology, Ljubljana, Slovenia; Faculty of Medicine, University of Ljubljana, Ljubljana, Slovenia; Jesenice General Hospital, Jesenice, Slovenia; Breast Center, University Hospital Basel, Basel, Switzerland; Cancer Center Baselland, Cantonal Hospital Baselland, Liestal, Switzerland

**Keywords:** breast cancer, chemotherapy, quality of life, cognitive dysfunction, fatigue

## Abstract

**Background:**

We aimed to identify changes in quality of life after breast cancer treatment and compare them with the normative population data for the Slovenian population.

**Patients and methods:**

A prospective, single-group, cohort design was used. A total of 102 early breast cancer patients treated with chemotherapy at the Institute of Oncology Ljubljana were included. Of those, 71% returned the questionnaires after one-year post-chemotherapy. The Slovenian versions of the European Organisation for Research and Treatment of Cancer (EORTC) QLQ C30 and BR23 questionnaires were used. Primary outcomes were a comparison of global health status/quality of life (GHS) and C30 Summary Score (C30-SumSc) at baseline and one-year post-chemotherapy with the normative Slovenian population. The exploratory analysis evaluated the differences in symptoms and functional scales of QLQ C-30 and QLQ BR-23 between baseline and one-year post-chemotherapy.

**Results:**

At baseline and one-year post-chemotherapy, C30-SumSc of patients was lower than the predicted C30-SumSc from the normative Slovenian population by 2.6 points (p = 0.04) and 6.5 points (p < 0.001), resp. On the contrary, GHS was not statistically different from predicted either at baseline or after one year. Exploratory analysis revealed that one-year post-chemotherapy compared to the beginning of chemotherapy, patients had statistically significantly and clinically meaningful lower scores in body image and cognitive functioning, and increased symptom scores for pain, fatigue, and arm symptoms.

**Conclusions:**

The C30-SumSc is reduced one-year post-chemotherapy. Early interventions should be directed toward the prevention of the decline of cognitive functioning and body image, and to alleviate fatigue, pain, and arm symptoms.

## Introduction

Global cancer statistics for 2020 estimated 2,261,419 new cases of breast cancer worldwide, which represents 11.7% of all cancers. It became the most common cancer in humans, surpassing lung cancer incidence.^1^ Breast cancer survivors represent a large group of long-term cancer survivors with different health issues during and after treatment. According to Slovenian Cancer Registry data in 2019, breast cancer survivors (19.455), represent 14.3% of all cancer survivors (136.500).^2^ Half of the Slovenian breast cancer cases are diagnosed in women in the 20–65 age group, which means they are active in their professional careers and family life.

Patients with early breast cancer receive multi-modal cancer treatment (surgical and/or systemic treatment including chemotherapy, targeted therapy, and endocrine therapy, and/or radiotherapy). The treatment they receive greatly affects their quality of life (QoL). For example, surgery and radiation therapy could cause local side effects, like breast and arm pain, and arm lymphoedema, however, systemic therapy could have numerous acute or long-lasting systemic side effects (nausea, neuropathy, cardiotoxicity, fatigue, cognitive dysfunction etc.). Thu s, comprehensive cancer therapy affects many functional or symptom scales of QoL. However, families and employers expect patients to recover fully in a short time.

Nowadays, QoL becomes also more and more important in terms of drug development. With the validated quality of life questionnaires (QLQ), specifically the European Organisation for Research and Treatment of Cancer (EORTC) core questionnaire (QLQ C30) and breast module (QLQ BR23)^3,4^, we can monitor the impact of treatment on patient-reported outcomes (PROs), like global health status/QoL and summary score, and compare different modules and symptoms over time.^5^ What is the QoL at the transition from treatment to survivorship where a woman is expected to be back to work? Arm symptoms and fatigue, as well as cognitive and physical dysfunction and work-related variables (e.g., physical demands at work), interfered with the ability to perform work. Schmidt *et al*. found associations between depressive symptoms, arm symptoms, lower education, and younger age with an impaired return to work after one year.^6^ Self-reported reasons that hinder the return to work were fatigue and cognitive problems.^6,7^ Additionally, social and personal factors influence the functioning and working ability of individuals.

Identifying dysfunctions that disable patients after breast cancer treatment and comparing QoL of patients with the normative population data^8,9^ could help caregivers provide survivors with more optimal care. They may benefit from specific interventions.

The aim of our study was to prospectively evaluate PROs in the cohort of early breast cancer patients at the start of chemotherapy and one year after the end of chemotherapy and to compare them with normative data for the Slovenian population. In our explorative analysis, we aimed to determine which functional and symptom scales appeared different one year after chemotherapy.

## Patients and methods

### Participants

Our current study cohort consisted of early breast cancer patients including all subtypes who had taken part in our previous prospective non-randomized cohort study evaluating the impact of mobile app use for symptom management on PROs during chemotherapy treatment. The inclusion criteria in the aforementioned study were patients with early breast cancer, treated with chemotherapy, possessing an Android-based smartphone for symptom reporting, and willing to fill in paper and pencil questionnaires reporting their quality of life while receiving treatment.^10^

In the current prospective study, we included 102 patients who had signed informed consent for the former study and were willing to fill in the additional QLQ C30, QLQ BR23, and socioeconomic questionnaires one year after the end of chemotherapy. Our first aim was to evaluate and compare pre-treatment and post-treatment PROs with normative data for the Slovenian population.^11^ This reference data on QLQ-C30 dimensions was obtained on 1231 healthy Slovenian individuals. Our second aim was to compare the post-treatment health-related quality of life (HRQoL) with the pre-treatment one. This data has not been reported yet.

### Study design

**FIGURE 1.**
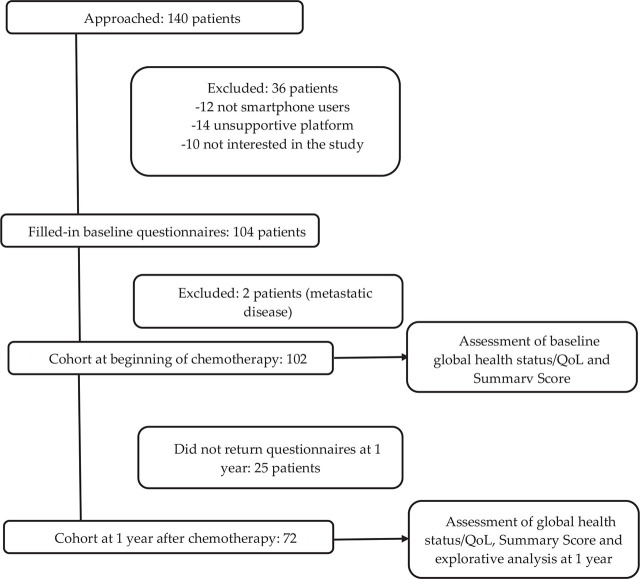
Consort diagram.

A prospective, single-group, cohort design was used combining data from a former two-arm trial with new follow-up data collected one year after the end of chemotherapy.^10^ Patients who were admitted for treatment with chemotherapy at the Institute of Oncology Ljubljana between December 2017 and September 2018 were eligible (Figure 1). Inclusion criteria were breast cancer stage I-III, treatment with neoadjuvant or adjuvant chemotherapy, and proficiency in using an Android-based smartphone. Exclusion criteria were stage IV breast cancer, a lack of mobile device proficiency or using non-Android-based smartphones, and not understanding Slovenian. In addition to chemotherapy treatment, patients were treated with anti-HER2 therapy (in case of HER2 positivity), surgery, endocrine therapy in case of hormone-receptor-positive disease, according to ESMO guidelines, and radiation therapy, if indicated.^12^ The patient's demographic characteristics and type of treatment were collected from patient charts. Ethical approval for this study had been obtained from the Ethics Committee of the Institute of Oncology Ljubljana (ERID-EK-43 and ERID-EK-0080/2019). All patients had given their written informed consent.

### Instruments

We used the Slovenian version of EOR TC QLQ C30 and QLQ BR23 questionnaires. QLQ C30 is a core questionnaire which includes 30 items, consisting of 5 functional scales (cognitive, emotional, physical, social and role functioning), 9 symptom scales, and two questions which include the patient's assessment of global health status/quality of life (GHS). QLQ BR23 has 23 questions comprising four functional scales (body image, sexual functioning, sexual enjoyment, future perspectives) and symptoms regarding the treatment of breast cancer (systemic therapy side effects, arm and breast symptoms and others). Socio-economic questionnaires, used routinely for surveys at the Slovenian Cancer Registry, included questions about age, gender, social class, employment, marital status, education, and place of residence.

### Outcome measures

Our primary outcomes were GHS and C30 Summary Score (C30-SumSc), derived from EORTC QLQ C30. Symptoms and functional scales of QLQ C30 and QLQ BR23 were used in the exploratory analysis only. The EORTC Quality of Life Scoring Manual was followed.^13^ All scales had values from 0 to 100, where 100 represented the best GHS, the best functioning, or the worst symptoms. The C30-SumSc, which ranged from 0 (worst) to 100 (best), was calculated from 13 out of 15 EORTC QLQ C30 scales (the GHS and financial difficulties scale were excluded) in accordance with Giesinger *et al*. and instructions from the EORTC.^5,14^ The patient's assessment of the clinical significance of changes in QLQ C30 and QLQ BR23 scores were interpreted as “slight” change either for better or for worse when the mean change in scores was about 5 to 10 points; “moderate” change for about 10 to 20 points; and “severe” change greater than 20 points. An established threshold for a clinically meaningful difference in QoL was previously set to 10 points.^15^

Data for GHS and C30-SumSc data for the normative Slovenian population were obtained from recently published work by Velenik *et al*.^11^ We computed each patient's predicted normative values for C30-SumSc and GHS (co-primary outcomes) according to this external reference.^11^ Using the patient's age (categorized as 18–39, 49–59, 60+) and self-rated social class (lower, middle, higher), the predicted normative value represented the patient's scores if she had not had cancer.

### Statistical analysis

Categorical variables were summarized with frequencies and percentages. Numerical variables were described with means and standard deviations (or medians and interquartile ranges if distributions were asymmetric).

We compared the mean C30-SumSc and GHS of our patients with the normative general Slovenian population.^11^ The mean C30-SumSc and GHS at the start of chemotherapy and one-year post-chemotherapy were compared with the corresponding mean of the normative values using two-tailed one-sample t-tests as the variability for the normative values could not be considered (the normative values were computed from the estimates from the article,^11^ standard errors of the estimates were not reported).

As a part of the exploratory analysis, we performed a comparison of the GHS, C30-SumSc and QLQ C30 and QLQ BR23 scales between inclusion (start of chemotherapy) and after one year in a smaller group (72 patients) that had available data on both times. For statistical comparison of scales based on at least two questions, we calculated the average difference and 95% confidence intervals ([After 1 year] – [At inclusion]) with two-tailed paired t-tests and 95% confidence intervals (CIs). For scales based on one question, we performed Wilcoxon's test of predicted ranks.

For all scales but C30-SumSc and GHS (primary outcomes), the corresponding p-values were adjusted using the Holm method to control the family-wise error rate as so many hypotheses were tested. Corrected p-values allow a conclusion per population, but uncorrected ones do not.

An (adjusted) p-value smaller than 0.05 was considered statistically significant. Analyses were performed using R statistical software (version 3.6.3)^16^ and SPSS v.24.0 (IBM Corporation).

## Results

### Participants

At the beginning of chemotherapy, we included 102 patients (Table 1, left column). These patients were compared with the normative Slovenian population regarding baseline GHS and C30-SumSc. Seventy-two patients (71%) returned questionnaires at one-year post-chemotherapy (Table 1, right column). The median age of this cohort was 51.5 years. 44% of patients were T1 and 43% were node-negative. Regarding subtype, 49% were luminal B-like, 19% luminal HER2+, 8% HER2+ nonluminal, 14% were triple negative and 10% were luminal A-like. The most common type of surgery was breast-conserving surgery with sentinel node biopsy (39%). All patients were treated with chemotherapy (adjuvant (69%) or neoadjuvant (31%)), 28% had anti-HER2 treatment, and 79% had adjuvant endocrine therapy. 82% of patients received adjuvant radiation therapy. Patients’ socioeconomic characteristics are available in Supplementary Table 1. For the calculation of predicted normative values of GHS and C30-SumSc, gender, age, and social class were used, according to Velenik *et al.*^11^ Their model was based on 1231 persons, of them 612 (49.7%) were females. The age distribution of females was: 30.7% in cohort 18–39 years, 42.8% in cohort 40–59 years, and 26.5% in cohort 60–90 years. Self-rated social status of females was: 30.9% belonged to the lower, 57.4% to the middle, and 11.8% to the upper social class.^11^

**TABLE 1.** Clinical characteristics of participants at beginning of the study and after one-year post-chemotherapy

**Characteristic**	**At inclusion-before chemotherapy (n = 102) n (%)**	**One year after end of chemotherapy (n = 71) n (%)**
Tumour stage		
T1	6 (45.1)	32 (44.4)
T2	43 (42.2)	33 (45.8)
T3	13 (12.7)	7 (9.7)
Lymph node stage		
N0	43 (42.2)	31 (43.1)
N1	38 (37.3)	30 (41.7)
N2	12 (11.8)	9 (12.5)
N3	9 (8.8)	2 (2.8)
Tumour subtype		
Luminal A-like	12 (11.8)	7 (9.7)
Luminal B-like	47 (46.1)	35 (48.6)
Luminal B HER2 positive	19 (18.6)	14 (19.4)
HER2 positive	8 (7.8)	6 (8.3)
Triple-negative	16 (15.7)	10 (13.9)
Type of surgery		
Breast-conserving surgery + Sentinel node biopsy	37 (36.3)	28 (38.9)
Breast-conserving surgery + Axillary dissection	14 (13.7)	11 (15.3)
Mastectomy + Sentinel node biopsy	23 (22.5)	15 (20.8)
Mastectomy + Axillary dissection	28 (27.5)	18 (25.0)
Breast reconstruction		
None	78 (76.5)	55 (76.4)
Deep inferior flap	13 (12.7)	10 (13.9)
Tissue expander, followed by silicone implant	11 (10.8)	7 (9.7)
Chemotherapy type		
Anthracyclines and taxanes	70 (68.7)	51 (70.8)
Anthracyclines only	19 (18.6)	13 (18.1)
Taxanes only	10 (9.8)	6 (8.3)
CMF	3 (2.9)	2 (2.8)
Anti-HER2 therapy	27 (26.4)	20 (27.8)
Adjuvant endocrine therapy	78 (76.5)	57 (79.2)
Radiotherapy	81 (79.4)	59 (81.9)

CMF = cyclophosphamide, methotrexate, fluorouracil; HER2 = human epidermal growth factor receptor 2

### Primary outcomes

**FIGURE 2.**
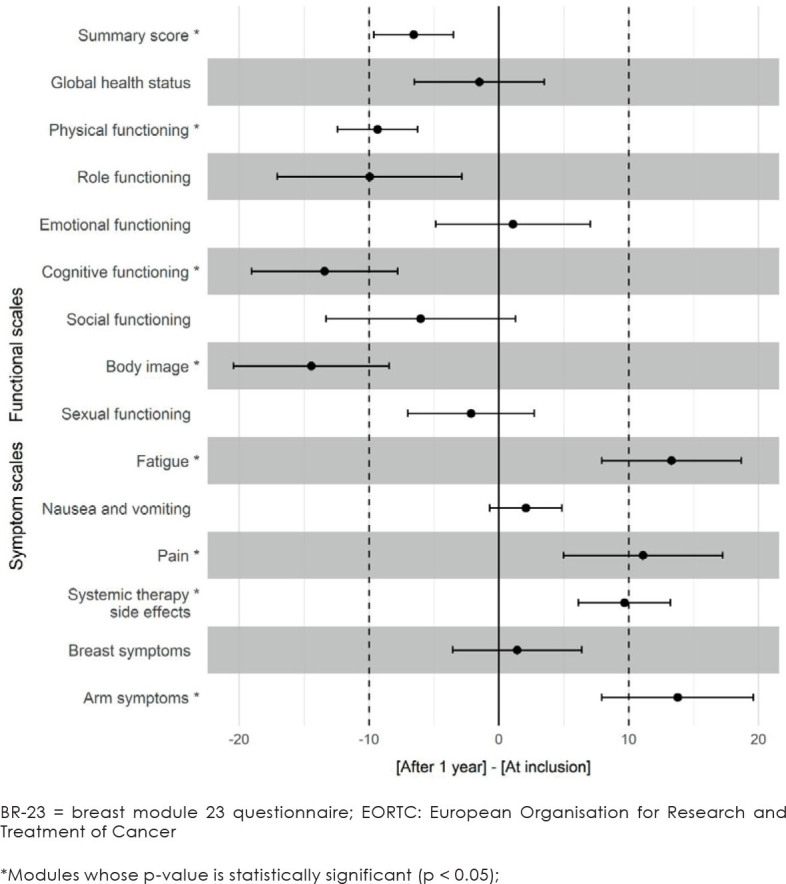
Difference of the global health status/quality of life (GHS), C-30 Summary Score (C-30 SumSc), and functional and symptoms scales of EORTC C-30 and BR-23 questionnaires.

At inclusion (before the start of chemotherapy), C30-SumSc of our patients was statistically significantly lower than the predicted C30-SumSc in the general Slovenian population, namely by 2.6 points (p = 0.04). After one year, compared to the start of chemotherapy, patients’ mean C30-SumSc decreased by 6.5 points, which was statistically significant (p < 0.001), from the patient perspective as a slight change for worse, but not clinically meaningful change (Table 2, Figure 2 - first line). On the contrary, GHS was not statistically or clinically significantly different from predicted either at inclusion or after 1 year (Table 2, Figure 2 - second line).

**TABLE 2.** Patient-reported outcomes presented by EORTC C30 Summary Score (C-30 SumSc) and global health status/quality of life (GHS) at inclusion (beginning of chemotherapy), one-year post-chemotherapy, and the difference among both times

		**Predicted**	**At inclusion**		**At 1 year**		**[1 year] - [At inclusion]**	
	n	mean	Mean (95% CI)	p	Mean (95% CI)	p	Mean (95% CI)	p
C-30 SumSc	70	90.9	88.5 (86.1, 90.8)	0.04	82 (78.4, 85.5)	< 0.001	−6.5 (−9.6, −3.4)	< 0.001
GHS	71	72.7	71.1 (66.7, 75.6)	0.50	69.6 (65, 74.2)	0.19	−1.5 (−6.6, 3.5)	0.55

EORTC = European Organisation for Research and Treatment of Cancer

### Exploratory analysis

The exploratory analysis (Figure 2) revealed that patients had significantly lower functioning one year after chemotherapy compared to the beginning of chemotherapy in 3 functional scales: in body image and cognitive functioning, the difference in our sample was > 10 points (clinically meaningful change), and in physical functioning under 10 points (clinically not meaningful). Similarly, pain, fatigue, and arm symptoms score significantly increased by more than 10 points (clinically meaningful), and systemic therapy side effects increased by less than 10 points (clinically not meaningful).

## Discussion

At the start of chemotherapy, early breast cancer patients had the same mean GHS as predicted from the general Slovenian population. C30-SumSc was statistically significantly lower, although this was not clinically significant since the difference was less than 10 points.

GHS in breast cancer patients one year after the end of chemotherapy was on average as good as before the beginning of chemotherapy. C30-SumSc was statistically significantly worse, from the patient's perspective as a slight change for worse, although deterioration of 6.5 points is not considered clinically important. In the e xploratory analysis, we found significant deterioration in some functional scales and increased symptoms in breast cancer patients one-year post-chemotherapy compared to the pre-chemotherapy state. Cognitive functioning, body image, and physical functioning significantly deteriorated. Among symptoms, increased arm symptoms, pain, fatigue, and systemic therapy side effects were self-reported (Figure 2).

Patients in our study did not perceive deterioration of GHS by a cancer diagnosis or cancer treatment. That means that patients perceived good overall well-being, as they had not been ill. A similar finding for GHS in patients 1–15 years post-diagnosis was found by others.^7,8,9,17^ On the other hand, our patients reported a slight deterioration of C30-SumCs one-year post-chemotherapy, probably due to the toxicities of multimodality treatment. Ferreira *et al*. noted similar persistent deterioration of C30-SumSc after two years in premenopausal patients treated with chemotherapy and postmenopausal patients treated with endocrine therapy.^18^ Deterioration of C30-SumSc is not only due to treatment but could be also due to the progression of cancer. C30 Sum-Sc has recently been shown as an independent prognostic factor for overall survival in several cancers.^19^

In our explorative analysis, we found a detrimental effect of cancer treatments (either chemotherapy, endocrine therapy, surgery, or radiotherapy) on specific functional and symptom scales, evaluated with QLQ C30 and QLQ BR23. Patients reported clinically meaningful deterioration in cognitive functioning and body image from baseline to one-year post-chemotherapy. Deficits in cognitive, role, social, and emotional functioning, particularly in young patients, were also reported by others 1–10 years after surgery.^8,20,21^ Compared to the general population, researchers found significantly lower mean scores for cognitive and social functioning, role functioning and emotional functioning, physical functioning and body image, and future perspective between 5–15 years post-therapy.^7,9,17,22^ That means that the consequences of treatment could be life-long. Interestingly, some serum markers of systemic inflammation were found to be statistically significantly higher in cancer survivors treated with chemotherapy even 20 years after chemotherapy and were associated with lower cognitive performance.^23^ Breast reconstruction, however, improved physical functioning and body image compared to breast-conserving surgery; the same applies to social functioning and future perspective.^24,25^

In addition to detrimental effects on functional scales, our exploratory analysis showed significantly increased symptom scores after one year compared to baseline: fatigue, arm symptoms, pain, and, to a lesser degree, the systemic therapy side effects. In addition to these symptoms, other researchers reported insomnia or sleep disturbances, breast symptoms and financial difficulties, dyspnoea, hot flashes, sexual problems, and polyneuropathy.^7,8,9,17,20^ Some differences in symptoms, however, were described only as trivial, with small clinical relevance.^17^

We suppose that pain and arm symptoms are related to higher nodal burden and consequently performed axillary dissection and irradiation. However, perceived fatigue, pain, and arm symptoms could also reflect less personal engagement in avoiding or managing these symptoms. Sixty-nine percent of our patients had primary surgery, followed by adjuvant chemotherapy, and 31% had the opposite sequence of treatment. In view of this information, we would expect that arm symptoms (from the BR-23 questionnaire) will be greater at the beginning (at inclusion in the study) than 1-year post-chemotherapy. But it turned out the opposite. We can explain this finding by the fact that arm symptoms are scores from three items, namely pain (not only pain in the arm, but also pain in the shoulder), swelling of the arm, and difficulties in the mobility of the arm. The swelling of the arm usually occurs with a delay. Swelling is more common when axillary dissection is performed (in our case 35.3% of patients) than when removing only sentinel lymph nodes. Radiotherapy, which was delivered to 81.9% of our patients, could contribute to swelling and pain as well.

Symptom pain of the C30 questionnaire is about pain anywhere and is made up of two questions, whether the pain is present and the question if it affects every day functioning. Generally, chemotherapy, especially taxanes, received by 79.1% of our patients, also contributes to the pain. Sensory polyneuropathy, not only hurts but often impedes normal functioning (walking, fine motoric). Additional pain could be contributed to adjuvant endocrine therapy (tamoxifen mainly affects large joints, and aromatase inhibitors affect small joints). Yoon *et al*. found variation in symptom reporting influenced by race/ethnicity and other sociodemographic characteristics, and several comorbid conditions.^26^

Returning to work is a significant milestone for breast cancer survivors.^7^ Von Ah *et al*. found that everyday cognition correlates with work engagement. What do these findings mean for cancer survivors in the setting of clinical practice? Cognitive dysfunction and fatigue are the most important issues for patients, especially if they are employed.

Cognitive dysfunction after chemotherapy (“chemo brain”) is described as the impairment of memory, attention, executive functions, and processing speed.^27,28^ Recently it has also been reported for hormonal therapy (tamoxifen and nonsteroid aromatase inhibitors), targeted therapy, immunotherapy, and due to cancer itself, combined in terminus “cancer-related cognitive dysfunction”.^28^ Subjective cognitive problems were reported by half of breast cancer patients after chemotherapy, but only 15–25% had an objective cognitive decline.^28^ Despite the mild-to-moderate severity of cognitive dysfunction, it represents an important issue for patients.^27^ It is especially true for patients who are employed.^7,8,26^ Impaired cognitive functioning in our patients one-year post-chemotherapy could be related to treatment with chemotherapy and surgery (general anesthesia) as well as adjuvant endocrine treatment (85% of patients).

Among symptoms, fatigue is reported most regularly in all studies. Fatigue is a subjective feeling of lack of energy, of physical, emotional, and/or cognitive tiredness or exhaustion related to cancer and/or cancer treatment, and interferes with usual functioning.^29^ It is a multidimensional symptom that accompanies patients while receiving chemotherapy and can last many years after chemotherapy.^27^ As with cognitive dysfunction, it could be associated with the type of treatment (surgery, chemotherapy, endocrine therapy, targeted therapy, or radiotherapy).

The rehabilitation of cancer survivors should be diverse, according to the needs of the individual patient. For example, physical functioning and fatigue could be improved with regular exercise.^30^ Arm symptoms could be managed with physical rehabilitation and more specifically lymphoedema treatment, elastic compressive gloves, and pain-killers. Pain (in the breast, arm, joints, peripheral polyneuropathy) should be appropriately managed by a pain specialist. Cognitive rehabilitation could include cognitive rehabilitation programs, physical activity, or relaxation programs.^27^ Hot flashes and sexual issues could be managed by a gynecologist. With fewer symptoms and better symptom scales patients would probably have better cognitive and role functioning and a better body image. In order to improve the QoL of cancer survivors, a pilot study on the comprehensive rehabilitation of breast cancer patients is underway at our institute. Identifying problems early probably allows an earlier targeted approach, thus leading to better patient functioning, an earlier return to work, and less absenteeism in the workplace. Evaluation of the results of comprehensive rehabilitation on improving functional and symptom scales is eagerly awaited.

### Strengths of the study

Firstly, this is a prospective cohort study of health-related quality of life, using validated questionnaires and tools, such as GHS and C30-SumSc, recommended by the EORTC. Many studies perform only cross-sectional data analysis. Secondly, we performed a comparison with our normative population to obtain information about what patients’ scores would be without cancer.

### Limitations of the study

The first limitation is the small sample size and that we did not have a baseline value of items in the questionnaires before any cancer treatment. Secondly, we included only patients that were proficient in using smartphones, and thus probably inadvertently chose a subset of the population that is highly motivated, healthier, and with middle social status. However, those patients were supported by a mobile app for coping with symptoms, which would probably be even heavier without the app. Thirdly, we included only Android-based smartphone users, which represented 80% of smartphones in Slovenia at that time. However, the app for IOS had not yet been made available. An additional weakness of our study is that the aspects of depression and anxiety, which affect cognitive functioning and fatigue, were not involved. Comorbidities were also not assessed – these are also significant predictors of symptoms, especially amongst those receiving chemotherapy.

## Conclusions

Patients with early breast cancer had similar GHS before chemotherapy as the normative Slovenian population, and it did not deteriorate with treatment. One year after chemotherapy, C30-SumSc deteriorated compared to that before chemotherapy. Early interventions should be directed toward the prevention of the decline of cognitive functioning and body image, and to alleviate fatigue, pain, and arm symptoms.

## Supplementary Material

Supplementary Material Details
