# Supplementary material for: Changes in the quality of life of early breast cancer patients and comparison with the normative Slovenian population
Source: Radiol Oncol. 2023 Jun 21;57(2):211–9. doi: 10.2478/raon-2023-0019 (PMC10286894; doi:10.2478/raon-2023-0019)
Supplement: Supplementary file 1 — Supplementary Material Details [file raon-2023-0019-sm.pdf]

sciendo Radiology and Oncology | Ljubljana | Slovenia | www.radioloncol.com
RADIOLOGY AND ONCOLOGY

Changes in the quality of life of early breast cancer patients and comparison with the normative Slovenian population

Cvetka Grasic Kuhar, Tjasa Gortnar Cepeda, Christian Kurzeder, Marcus Vetter

doi: 10.2478/raon-2023-0019

SUPPLEMENTARY TABLE 1. Patients' socioeconomic characteristics

Variable	n (%)
**Age category**	
18-39 years	13 (12.7)
40-59 years	69 (67.6)
60-90 years	20 (19.6)
**Marital status**	
Single	6 (8.5)
Divorced	5 (7.0)
In a stable relationship	7 (9.9)
Widowed	4 (5.6)
Married	50 (70.4)
**Education**	
Elementary school	6 (8.5)
High school	34 (47.9)
Professional school degree	14 (19.7)
College degree	13 (18.3)
Master's degree/doctorate	5 (7.0)
**Employment**	
Employed	48 (67.6)
Unemployed	3 (4.2)
Retired	16 (22.5)
Housewife/Farmer	4 (5.6)
Other	1 (1.4)
**Living environment**	
Urban	15 (20.8)
Suburban	20 (27.8)
Rural	37 (51.4)
**Social class**	
Lower	20 (28.2)
Middle	41 (57.7)
Higher	10 (14.1)
